# Supplementary material for: Structural snapshot of a bacterial phytochrome in its functional intermediate state
Source: Nat Commun. 2018 Nov 21;9:4912. doi: 10.1038/s41467-018-07392-7 (PMC6249285; doi:10.1038/s41467-018-07392-7)
Supplement: Supplementary file 1 — Supplementary Information [file 41467_2018_7392_MOESM1_ESM.pdf]

Structural snapshot of a bacterial phytochrome in its functional intermediate state

Andrea Schmidt, Luisa Sauthof, Michal Szczepek, Maria Fernandez Lopez et al.,

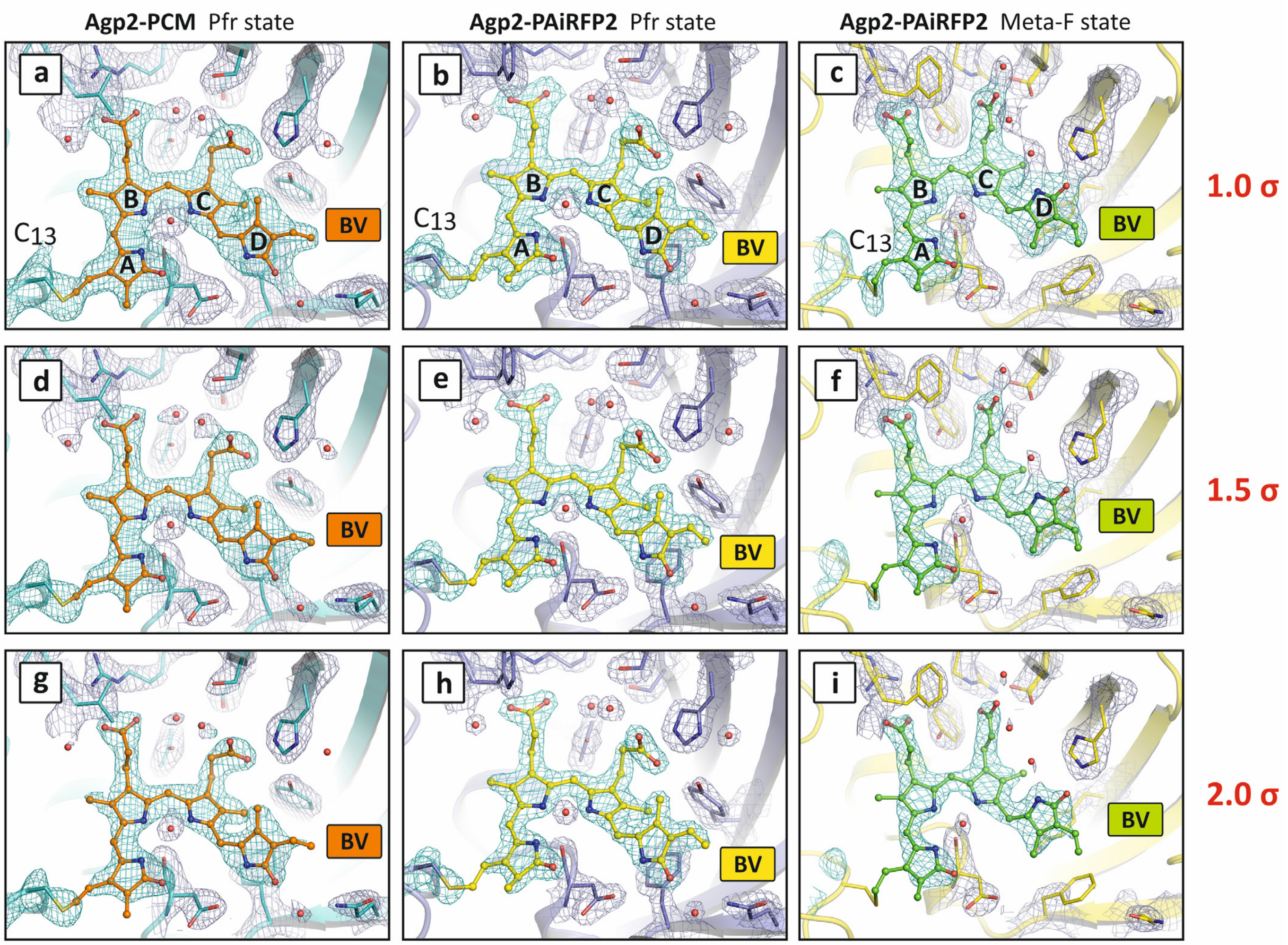

Supplementary Figure 1 | Quality of the 2*m*Fo-*D*Fc electron density of the chromophore binding pocket of Agp2-PCM and Agp2-PAiRFP2. Close-up view of the chromophore binding pocket of wild-type Agp2-PCM in the Pfr state (**a, d, g;** biliverdin (BV) in orange, protein in cyan; PDB entry 6G1Y), Agp2-PAiRFP2 in the Pfr state (**b, e, h**; BV in yellow, protein in violet; PDB entry 6G1Z) and illuminated Agp2-PAiRFP2 in a Meta-F sub-state (**c, f, i;** BV in green, protein in yellow; PDB entry 6G20, Mol A). All figures show the 2*m*Fo-*D*Fc electron densities of the chromophore (teal mesh) and the chromophore binding pocket (blue mesh) contoured at three different contour levels (1.0 (above), 1.5 (middle) and 2.0 σ (below)). BV, the protein backbone and selected amino acids are depicted in ball and stick, cartoon and stick representation, respectively; water molecules are shown as red spheres.

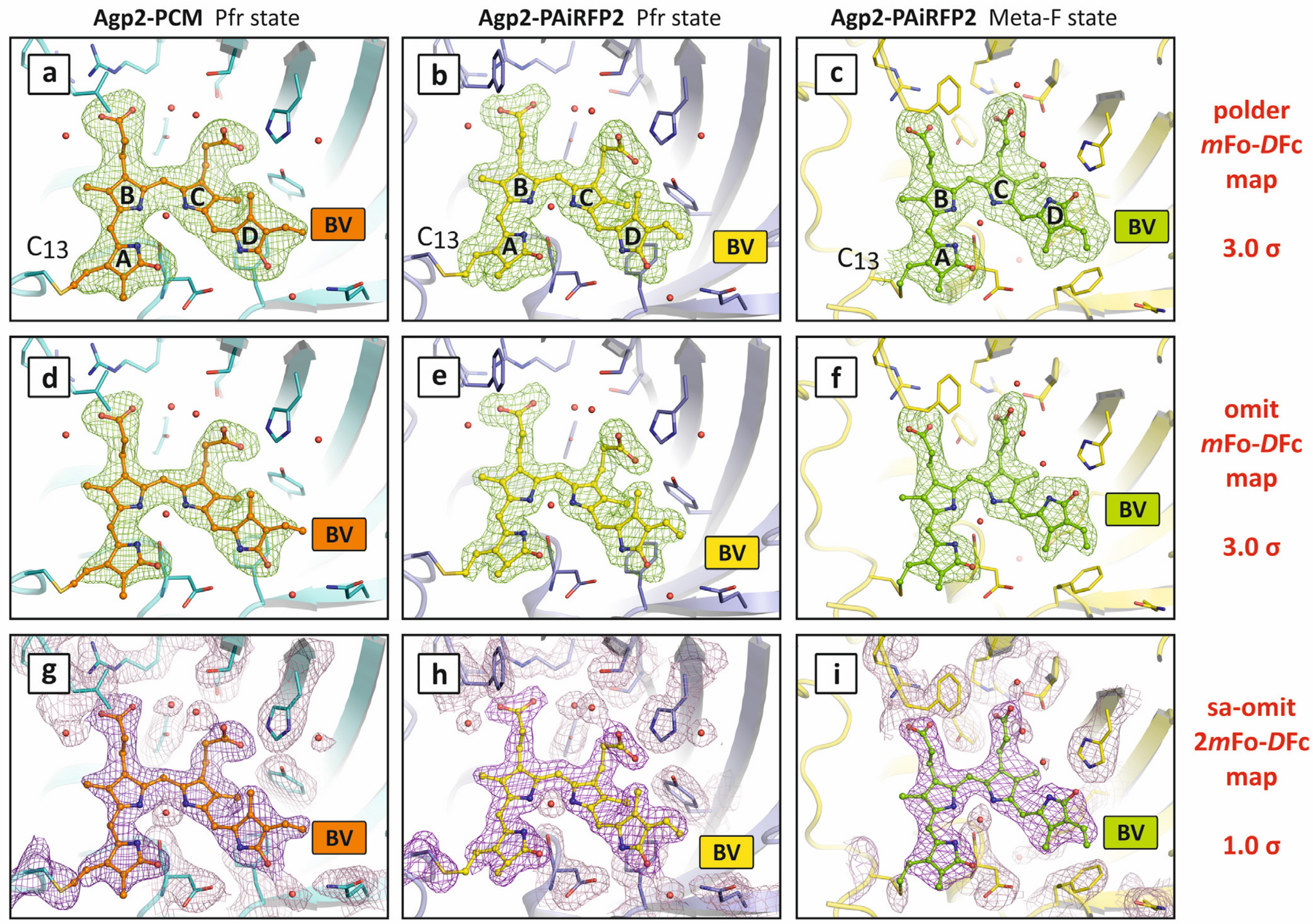

Supplementary Figure 2│ Different types of omit electron density maps of the chromophore of Agp2-PCM and Agp2-PAiRFP2. Close-up view of the chromophore binding pocket of wild-type Agp2-PCM in the Pfr state (**a, d, g;** BV in orange, protein in cyan; PDB entry 6G1Y), Agp2-PAiRFP2 in the Pfr state (**b, e, h;** BV in yellow, protein in violet; PDB entry 6G1Z) and illuminated Agp2-PAiRFP2 in the Meta-F sub-state **(c, f, i;** BV in green, protein in yellow; PDB entry 6G20, Mol A). Green meshes in panels **a-c** show the calculated "polder" *m*Fo-*D*Fc omit electron density map for BV contoured at 3.0 σ and calculated with the program phenix.polder[1]. BV was omitted in the calculation as well as bulk solvent around the ligand region. This procedure is expected to result in improved electron density in the ligand region that is not obscured by bulk solvent anymore. Figures **d-f** show *m*Fo-*D*Fc omit

electron density maps contoured at 3.0 σ. The BV chromophore was omitted in the map calculation. Figures **g-i** depict calculated "simulated annealing omit" 2*m*Fo-*D*Fc electron density maps (purple mesh) contoured at 1.0 σ. The BV chromophore was omitted in the calculation. For removing residual bias a simulated annealing step was included in the map calculations. The removal of phase bias is discussed in *e.g.* Pražnikar *et al.* 2009[2]. A "polder" map (**a-c**) is an omit map which excludes the bulk solvent around the omitted region[3]. A standard "omit" map (**d-f**) is generated by deleting part of the structure (here the bound BV) followed by re-calculating phases and $F_{(model)}$. This procedure is to remove phase bias by iterative (re)phasing from a modified $F_{(model)}$ or refinement of a modified model. A last and ultimate variant to remove the model bias is the "simulated annealing omit" map (**g-i**), which uses simulated annealing as an optimization method [e.g. 4].

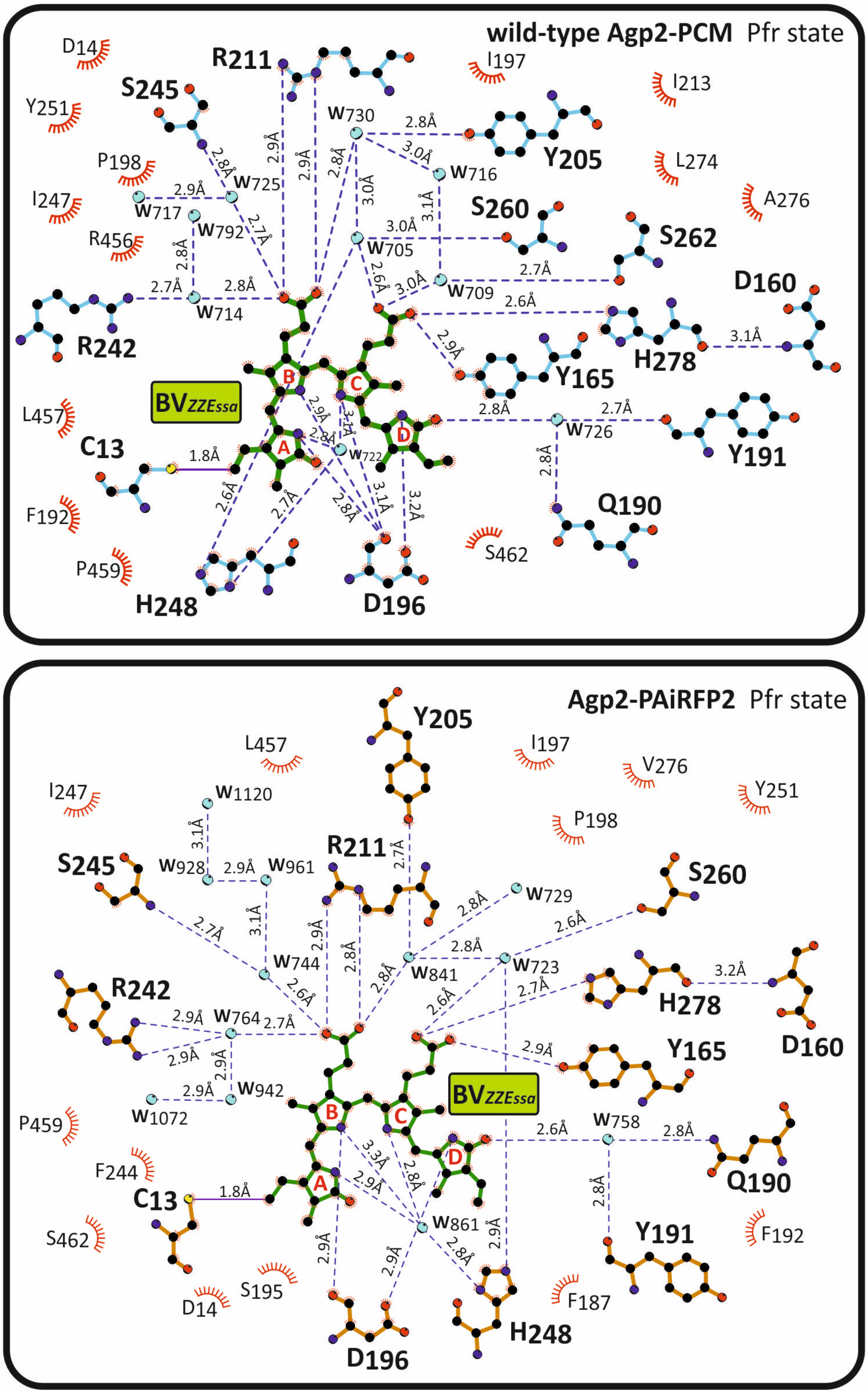

Supplementary Figure 3 | Interactions between biliverdin (green), the surrounding side chains (cyan/brown) and water molecules (cyan) of Agp2-PCM (Mol A; top figure) and

of Agp2-PAiRFP2 (Mol A; bottom figure) in the Pfr state (PDB entries 6G1Y and 6G1Z, respectively)**.** Potential hydrogen bonds and hydrophobic interactions were analysed using HBPLUS[5] with a maximum donor acceptor distance of 3.5 Å as implemented in the program LigPlot+ 1.45[6], which was also used to draw this schematic view. Potential hydrogen bonds are indicated by blue dashed lines and distance labels. Residues with closest distances of less than 4 Å are considered to be in van der Waals contact. These residues are shown as dashed, red segments of a circle.

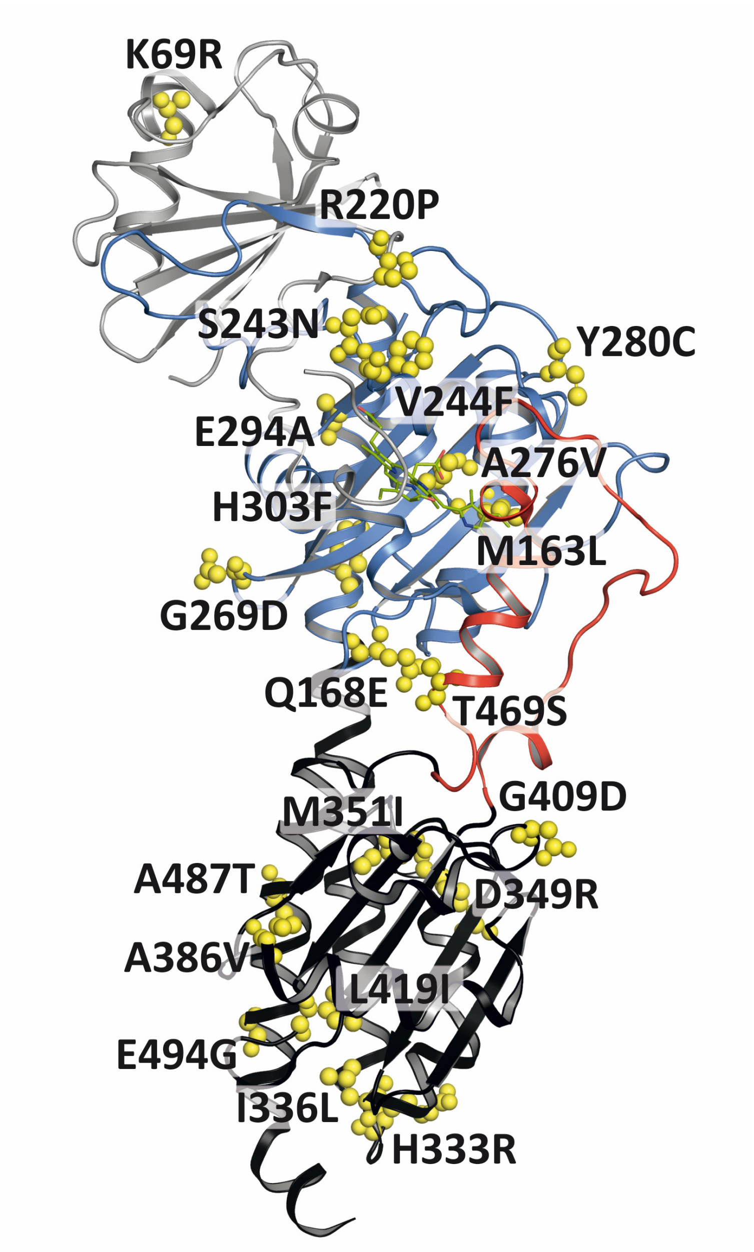

Supplementary Figure 4│ Overview of all substitutions in PCM of Agp2-PAiRFP2. Twenty one of the 24 substituted amino acids in the PCM of Agp2-PAiRFP2 (derived from the protein construct of Verkhusha and coworkers[7]) are highlighted as yellow spheres. The secondary structure of the PCM is visualized by showing α-helices and β-sheets in transparent ribbon representation. PAS, GAF and PHY domains are drawn in grey, blue, and black, respectively. The PHY tongue is highlighted in red. Three substituted amino acids at positions 83, 120 and 123 (substitutions R83K, G120D and A123T) are located in flexible loops of the crystal structure and could therefore not be resolved in the electron density map.

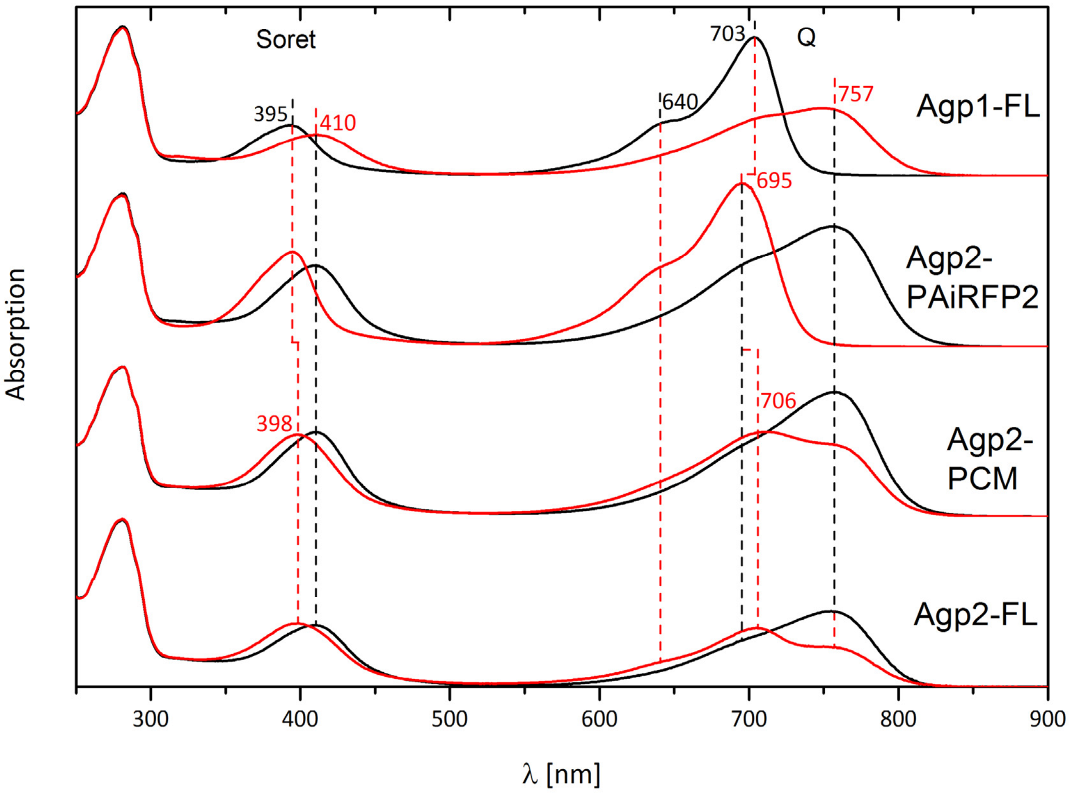

Supplementary Figure 5 │ UV-vis Absorption spectra of prototypical wild-type full-length Agp1 (Agp1-FL)[8], and the bathy phytochromes Agp2-PAiRFP2[7], wild-type Agp2-PCM and wild-type full-length Agp2 (Agp2-FL)[9]. Black traces display the absorption spectra of the dark adapted "parent" state. Red traces show the spectra after irradiation at the respective absorption maxima of the dark adapted states. The spectra show characteristic peaks and shifts between the dark adapted and the illuminated states. The Soret peak rises around 400 nm, whereas the Q-band maxima range from ca. 700 – 750 nm. Moreover, the illuminated states have different lifetimes. The final photoproduct of Agp2-PAiRFP2 is much longer stable (lifetime: 233 min)[7] than that of Agp2-PCM (20 s)[9] or Agp2-FL (170 s)[9]. Therefore, the Q-band shows a mixture of the Pr and Pfr state in the illuminated spectra of Agp2-PCM and Agp2-FL. The spectrum of the final photoproduct of Agp2-PAiRFP2 is characteristic of a protonated ZZZssa bilin chromophore as in the Pr or Meta-F states of phytochromes (cf. Agp1-FL, and the spectra of plant phytochrome in ref. 10)[10].

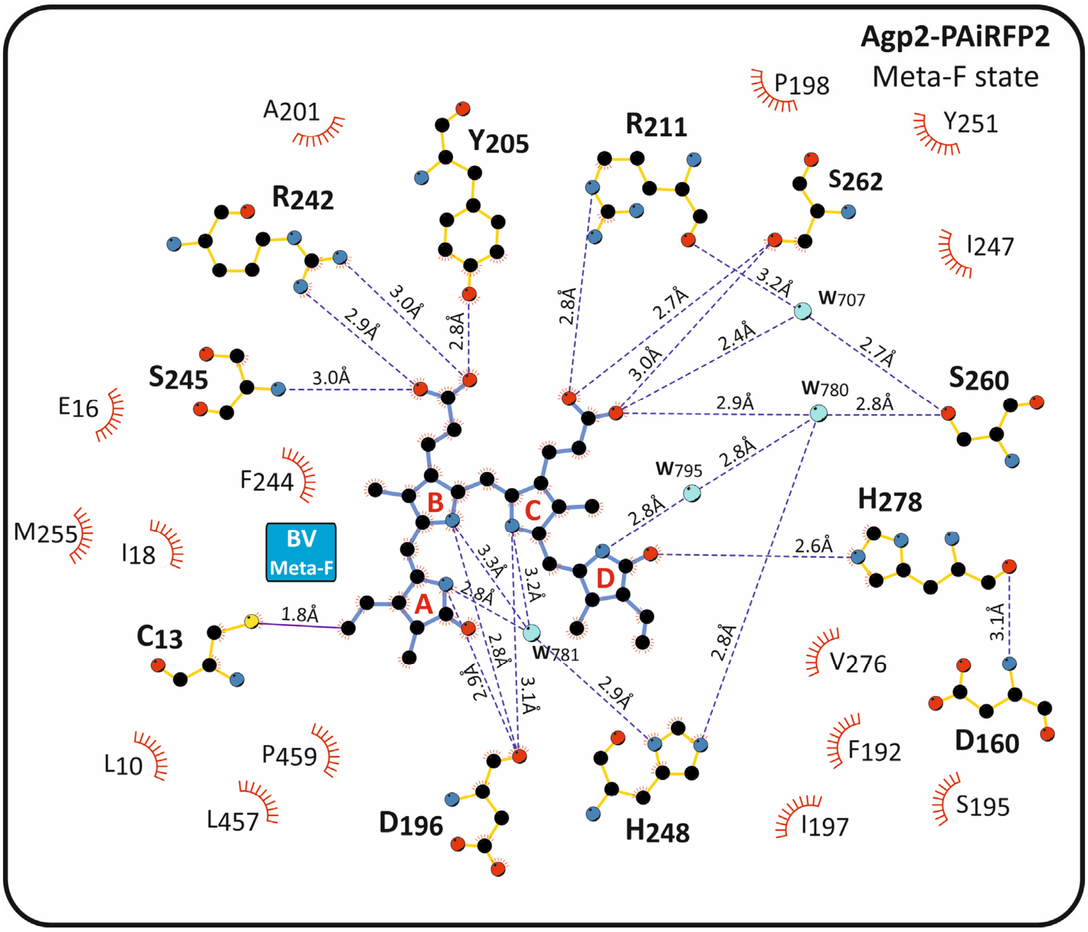

Supplementary Figure 6 | Interactions between biliverdin (blue) and the chromophore pocket of Agp2-PAiRFP2 (gold) in a light-induced Meta-F sub-state (PDB entry 6G20, Mol A)**.** Potential hydrogen bonds and hydrophobic interactions were analysed using HBPLUS[5] with a maximum donor acceptor distance of 3.5 Å as implemented in the program LigPlot+ 1.45[6], which was used to draw this schematic view. Potential hydrogen bonds are indicated by blue dashed lines and distance labels. Residues with closest distances of less than 4 Å are considered to be in van der Waals contact. These residues are shown as dashed, red segments of a circle.

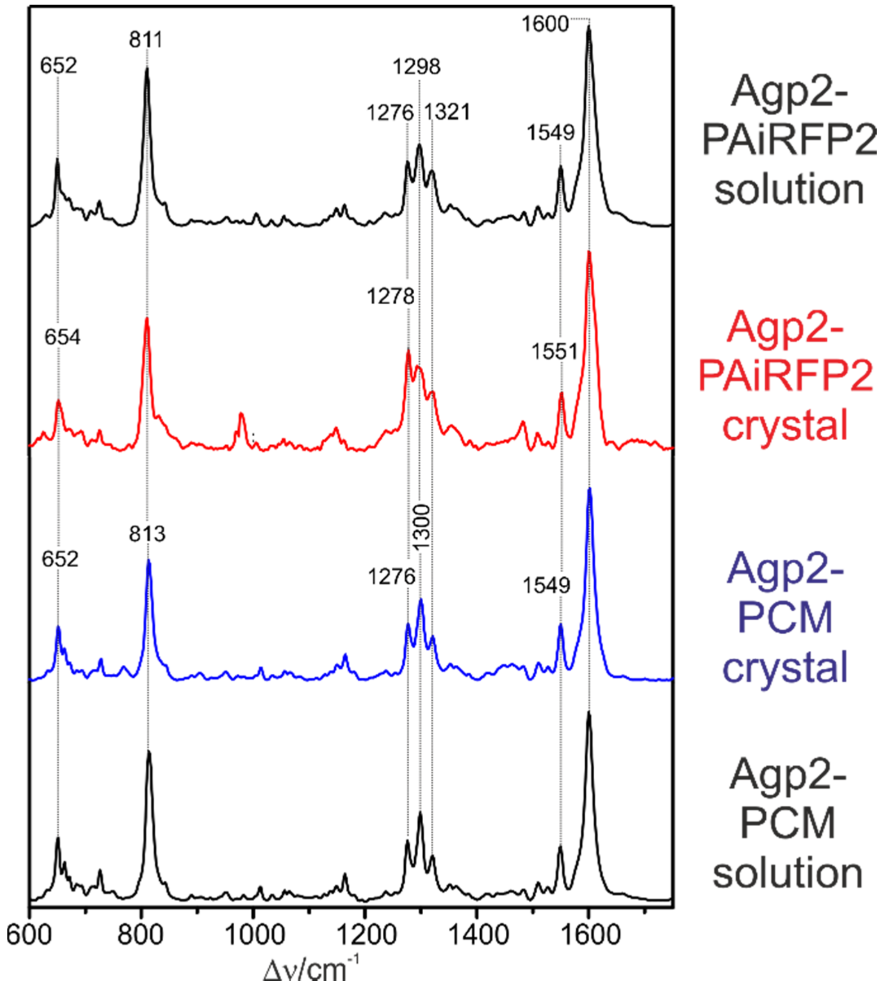

Supplementary Figure 7 | Resonance Raman spectra of the Pfr states of Agp2 variants from frozen solution and single crystals, measured at 90 K. Top black trace: Agp2-PAiRFP2 in frozen solution; red trace: Agp2-PAiRFP2 crystals; blue trace: methylated Agp2-PCM crystals; bottom black trace: Agp2-PCM in frozen solution. The spectrum of methylated wild-type Agp2-PCM crystals (blue trace) is identical to that of wild-type Agp2-PCM (bottom black trace) and wild-type full-length Agp2, each of both in frozen solution (not shown), demonstrating that methylation of Agp2-PCM at the protein surface, crystallization, and the lack of the output module do not affect the structure of the chromophore pocket. The spectra of Agp2-PAiRFP2 agree very well when comparing those taken from frozen solution (top black trace) and single crystals (red trace) implying that the inserted substitutions also do not alter the structure of the chromophore pocket. The differences between Agp2-PCM and Agp2-PAiRFP2

are very small and frequency deviations do not exceed ± 2 cm^{-1}, which is in line with the conclusions drawn from the comparative analysis of the crystal structures.

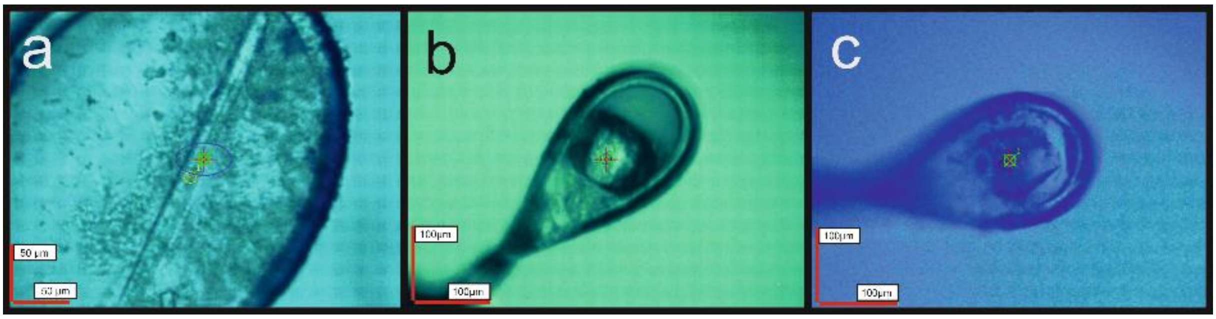

Supplementary Figure 8 │ Representative phytochrome crystals in cryo-loops of wild-type Agp2-PCM in the Pfr state **(a)**, Agp2-PAiRFP2 in the Pfr state **(b)** and light-induced Agp2-PAiRFP2 in the Meta-F state **(c)**. The crystals have been measured and datasets collected at the beamlines (**a**) ID30A-3[11], (**c**) ID23-1[12] at ESRF (Grenoble, France) and (**b**) BL14.1[13] at BESSY II (Berlin, Germany).

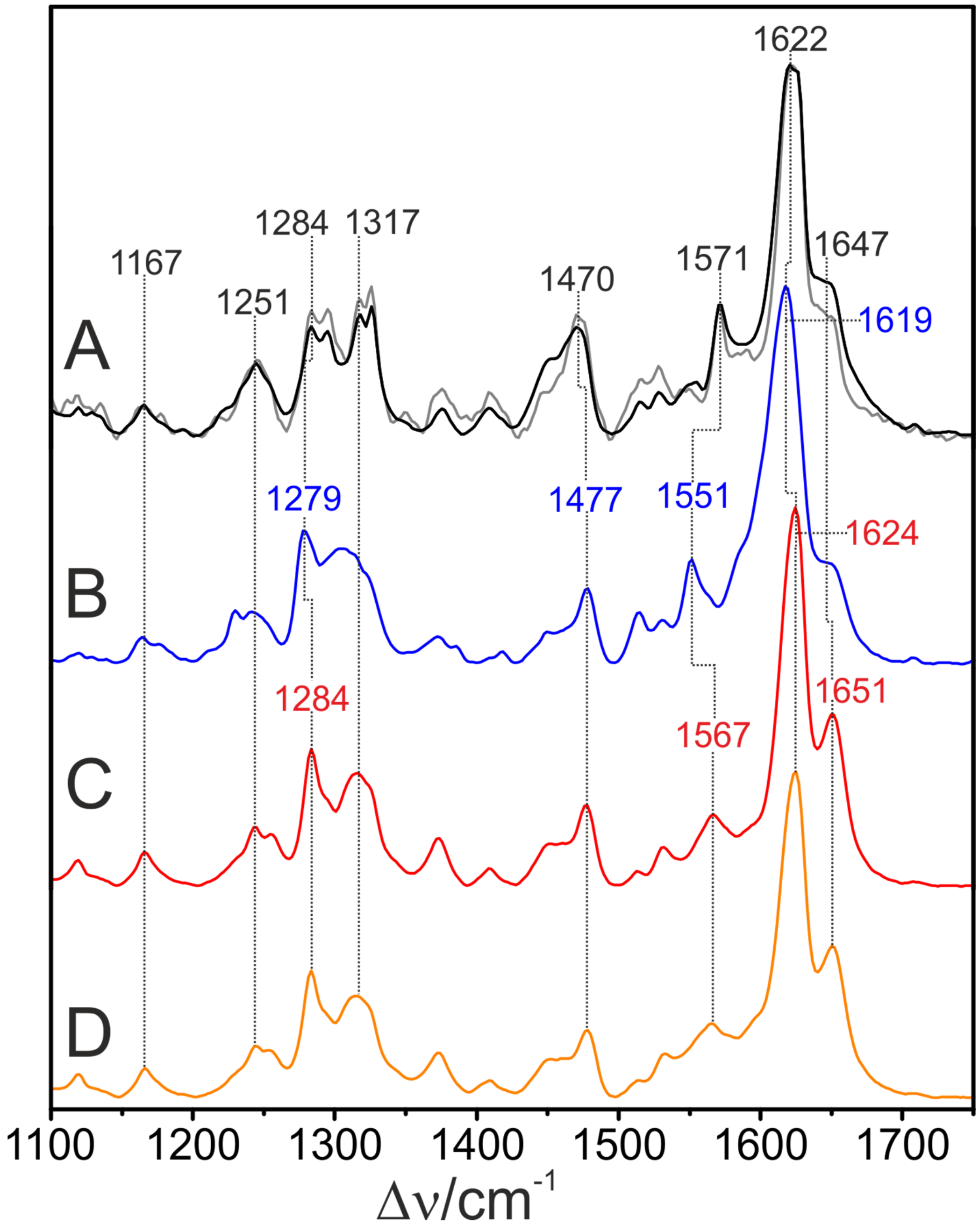

Supplementary Figure 9 │ Resonance Raman spectra of Agp2 variants in different states, measured in frozen solution (pH 7.8) at 90 K after irradiation at different temperatures. (**A**), Pr state of full-length wild-type Agp2, obtained after irradiation at 273 K (black); the grey trace corresponds to the spectrum after subtraction of the contribution of enol tautomer (spectra taken from[14]); (**B**), Meta-F state of wild-type Agp2-PCM (blue), obtained by irradiation at ca. 240 K; (**C**), Meta-F state of Agp2-PAiRFP2 (red), obtained by irradiation at ca. 240 K; (**D**), final

photoconversion product of Agp2-PAiRFP2 (orange), obtained by irradiation at 273 K. The spectra of Agp2-PAiRFP2 irradiated at 240 and 273 K (**C**, **D**) are identical, implying that chromophore relaxation is completed at ca. 240 K. For the wild-type Agp2-PCM, a reliable spectrum of the Pr state could not be obtained due to the fast thermal back reaction to Pfr at 273 K[15]. Thus, the photoconversion product obtained by irradiation at 240 K (**B**) is Meta-F of Agp2-PCM, which is identical to that obtained from the wild-type full-length Agp2 under the same conditions within the accuracy of the subtraction procedure[14], has to be compared with the final photoconversion product of the full-length wild-type Agp2 obtained upon irradiation at 273 K (**A**, Pr). Also in this case, we note far-reaching similarities in the overall band pattern but there are still some differences in frequencies and relative intensities between the Meta-F and Pr state. Among them the most notable differences refer to the C-D stretching (1622 *vs.* 1619 cm^{-1}) and the ring B/C N-H in-plane bending (1571 *vs.* 1551 cm^{-1}) which point to subtle differences in the C-D methine bridge geometry and different hydrogen bonding interactions of rings B and C. These alterations can only partly result from the enol-keto equilibrium that is formed in the Pr state of full-length wild-type Agp2 (see black and grey traces in **A**)[14] but indicate small additional conformational adjustments of the chromophore and its immediate surrounding during the Meta-F $\rightarrow$ Pr transition. Interestingly, the Meta-F intermediate of Agp2-PAiRFP2 (**C**) displays a RR spectrum that is very similar to the Meta-F spectrum of wild-type Agp2-PCM (**B**) but with the C-D stretching and N-H in-plane bending closer to those of the Pr state of full-length wild-type Agp2 (**A**). A possible explanation for these differences may lie in small changes in the hydrogen bonding network of the chromophore pocket that are reflected by the vibrational bands of the chromophore.

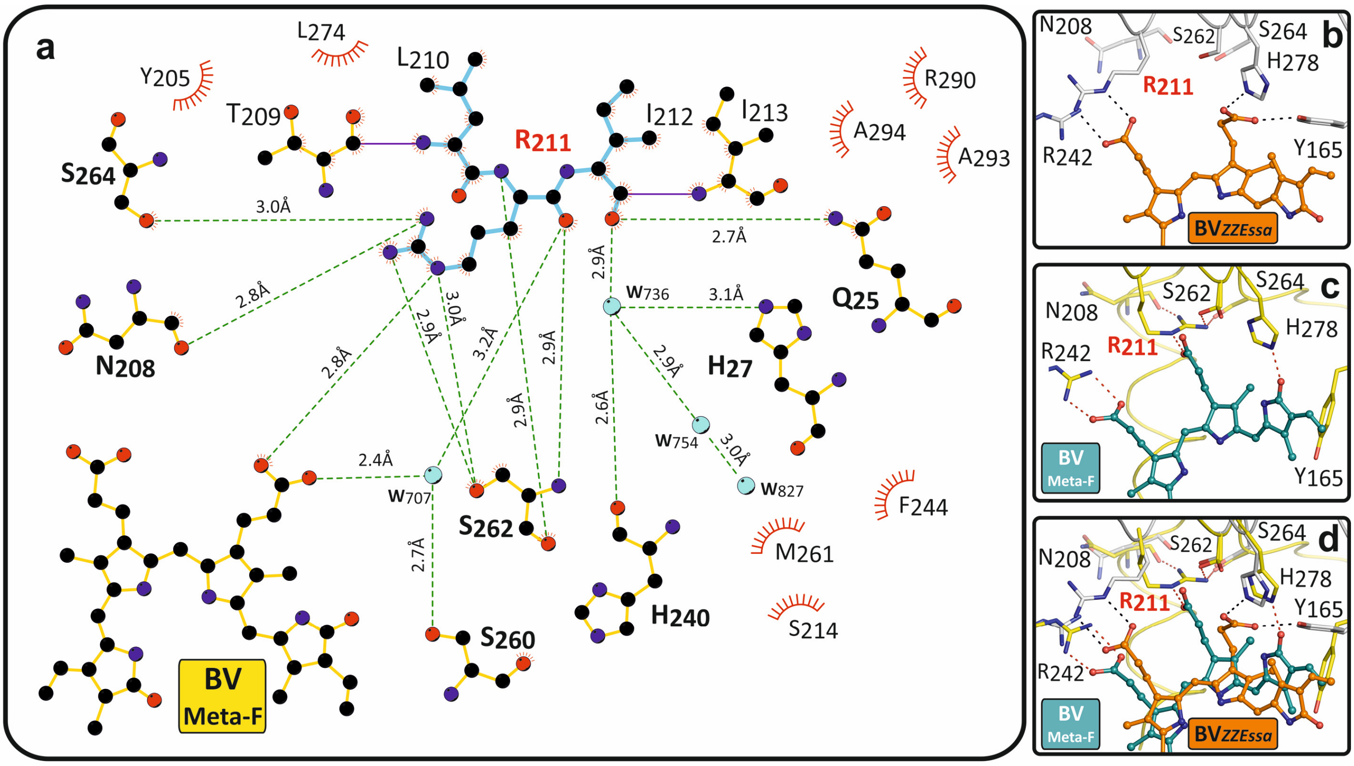

Supplementary Figure 10│ Interactions between moving Arg211 (blue) and the chromophore pocket of Agp2-PAiRFP2 (gold) in the light-induced Meta-F sub-state (Mol A). (a), potential hydrogen bonds and hydrophobic interactions were analysed using HBPLUS[5] with a maximum donor acceptor distance of 3.5 Å as implemented in the program LigPlot+ 1.45[6], which was used to draw this schematic view. Potential hydrogen bonds are indicated by green dashed lines and distance labels. Residues with closest distances of less than 4 Å are considered to be in van der Waals contact. These residues are shown as dashed, red segments of a circle. **(b-d)** Proteins are shown in ribbon representation, bilin-type chromophore in ball and stick representation and selected conserved amino acids around Arg211 are highlighted as sticks of **(b)** Agp2-PAiRFP2 in the Pfr state and **(c)** Agp2-PAiRFP2 in the Meta-F sub-state. A superposition of both states is shown in **(d)**. The Arg211 side chain rotates almost 180° from Pfr to Meta-F state and exchanges its binding partner from prop-B to prop-C of the BV chromophore. Instead of Arg211, Arg242 interacts in Meta-F with prop-B via a new salt-bridge.

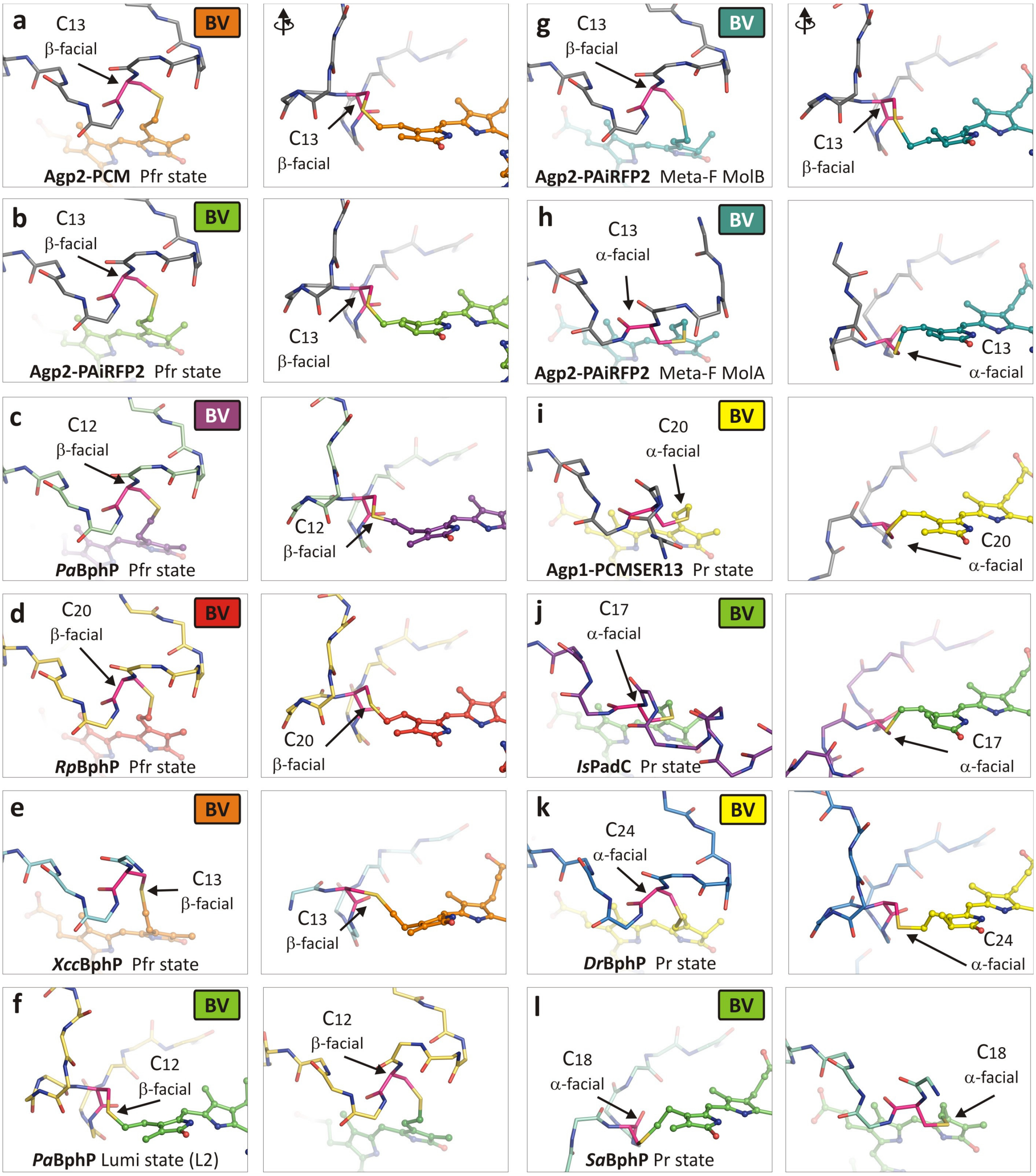

Supplementary Figure 11 | Orientation of cysteine binding to the bilin-type chromophore of different phytochromes (extension figure to Fig. 7). The protein backbone is represented as sticks and the chromophore in ball/stick representation. The binding cysteine is drawn in pink sticks; the attachment sides are always drawn in two

different views (left and right panel); (**a**) Agp2-PCM in Pfr state (PDB entry 6G1Y), (**b**) Agp2-PAiRFP2 in Pfr state (PDB entry 6G1Z), (**c**) *Pa*BphP in Pfr state (PDB entry 3NHQ)[16], (**d**) *Rp*BphP in Pfr state (PDB entry 4GW9)[17], (**e**) *Xcc*BphP in Pfr state (PDB entry 5AKP)[18], (**f**) *Pa*BphP in Lumi state (PDB entry 3NOT)[16], (**g**) Mol B of Agp2-PAiRFP2 in Meta-F sub-state (PDB entry 6G20), (**h**) Mol A of Agp2-PAiRFP2 in Meta-F sub-state (PDB entry 6G20), (**i**) Agp1-PCM$_{SER13}$ in Pr state (PDB entry 5HSQ)[19], (**j**) *Is*PadC in Pr state (PDB entry 5LLY)[20], (**k**) *Dr*BphP in Pr state (PDB entry 4Q0J)[21], (**l**) *Sa*BphP in Pr state (PDB entry 6BAF)[22]; all bacterial phytochromes reveal a consistent classification of α-facial cysteine binding in Pr state and a β-facial cysteine binding in Pfr state.

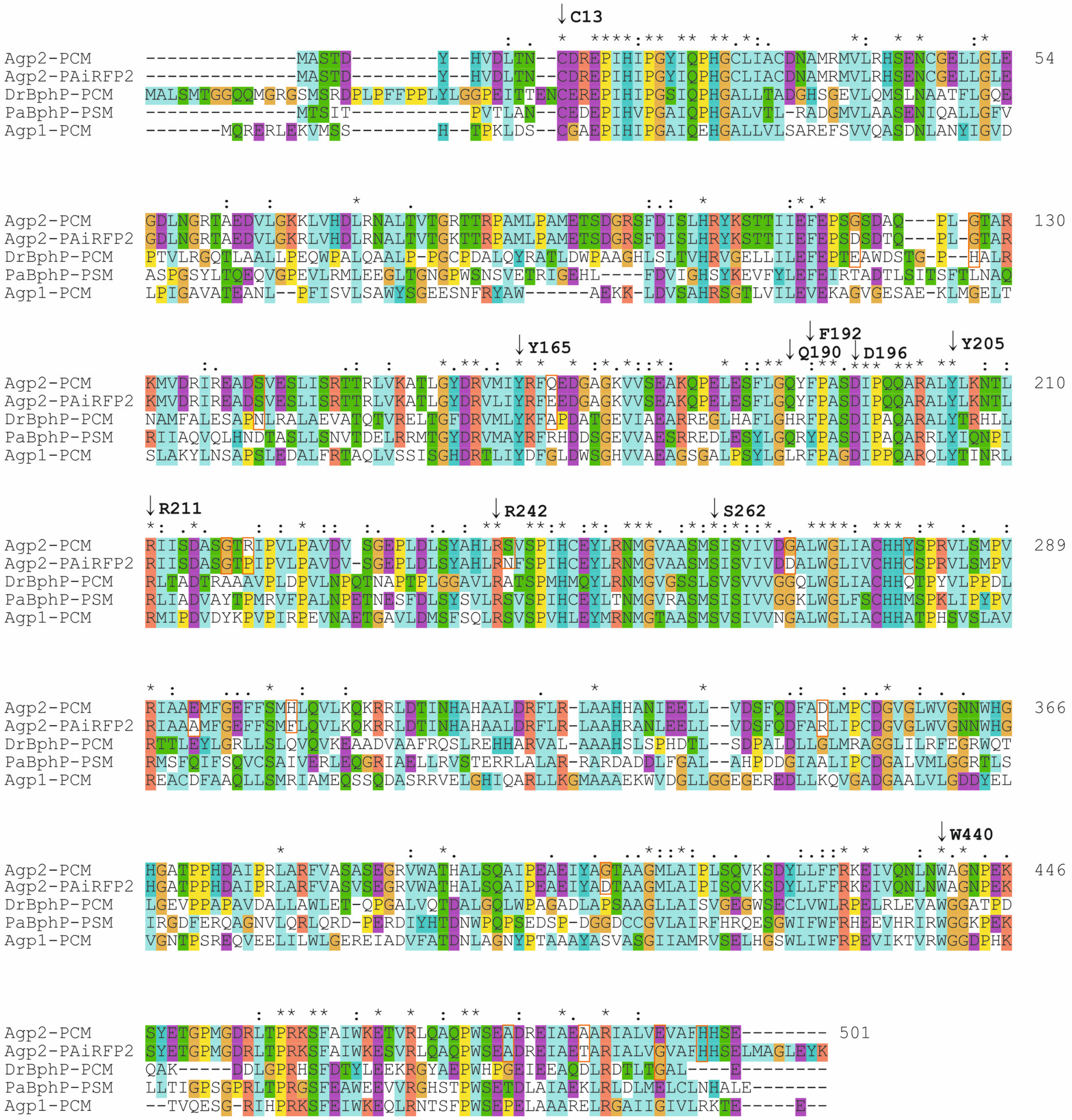

Supplementary Figure 12 │ Sequence alignment of the PCM – Photosensory Core Module (three N-terminal domains: PAS-GAF-PHY) of Agp2, Agp2-PAiRFP2, *Pa*BphP, Agp1 and *Dr*BphP. Shown are PCMs of bathy phytochrome Agp2-PCM (*Agrobacterium fabrum*, Uniprot ID: A9CI81) and Agp2-PAiRFP2 (*Agrobacterium fabrum*, Genbank ID: AGS83373.1), and *Pa*BphP-PCM (*Pseudomonas aeruginosa* Uniprot ID: Q9HWR3), and of

prototypical phytochromes Agp1-PCM (*Agrobacterium fabrum*, Genbank ID: AAT99575.1 and *Dr*BphP-PCM (*Deinococcus radiodurans*, Uniprot ID: Q9RZA4). The alignment was performed with the program Muscle (Version 3.8.31) using a gap penalty of 0.3 and default parameters. Conserved amino acids are labeled red for positively charged, magenta for negatively charged, green for polar, blue for hydrophobic residues, brown for Pro, yellow for Cys, orange for Gly[23].

Supplementary Table 1 │ Angles between the planes of neighbouring pyrrole rings of biliverdin Tilt angles between neighbouring pyrrole rings (as planes) of the biliverdin chromophores were determined using the program suite UCSF *Chimera*[24]. Please note that tilt angles determined by this method cannot distinguish between tilts that result from either clockwise or anti-clockwise rotation of two neighbouring rings. Equally, these angles cannot be used to distinguish between two different geometries where one ring is flipped within a plane. In order to obtain a more complete picture of the chromophore geometry, additional stereochemical information is required. The configuration and conformation of the biliverdin chromophore is indicated as either ZZZssa or ZZEssa for each structure. In all structures the chromophores adopt an α-facial disposition of their D-rings.

**Construct**	**wild-type Agp2-PCM** (PDB entry 6G1Y)	**Agp2-PAiRFP2** (PDB entry 6G1Z)	**Agp2-PAiRFP2** (PDB entry 6G20)	**Agp1-PCMSER13** (PDB entry 5HSQ)	**wild-type Agp1-PCM** (PDB entry 5I5L)
**state**	Pfr state<br>ZZEssa<br>Mol A / Mol B	Pfr state<br>ZZEssa<br>Mol A / Mol B	Meta-F state<br>ZZZssa<br>Mol A / Mol B	Pr state<br>ZZZssa<br>Mol A	Pr state<br>ZZZssa<br>Mol A
**A-B angle [°]**	17.0 / 17.7	23.2 / 27.3	15.6 / 30.9	7.8	6.6
**B-C angle [°]**	19.1 /20.8	19.2 / 19.5	7.6 / 12.7	11.2	7.2
**C-D angle [°]**	53.1 / 58.3	52.1 / 53.4	38.9 / 44.5	50.9	56.4

Supplementary Table 2 │ DNA Sequence

Construct	DNA Sequence
**Agp2-PCM**	cgaaatTAATACGACTCACTATAggGGAATTGTGAGCGGATAACAATTCCcctctagaaa<br>taattttgtttaactttaagAAGGAGatatacatATGGCATCCACGGACTATCATGTCGA<br>CCTGACCAATTGCGACCGCGAACCCATTCATATTCCAGGCTATATTCAACCGCATGGCTG<br>CCTTATCGCCTGCGATAACGCCATGCGAATGGTGCTGCGGCATTCCGAAAACTGCGGCGA<br>ACTGCTGGGACTTGAGGGCGATCTCAACGGCAGGACCGCCGAGGATGTGCTCGGCAAAAA<br>GCTCGTCCACGATCTCCGCAACGCGCTCACCGTCACCGGTAGAACCACGCGCCCCGCCAT<br>GCTGCCCGCAATGGAAACAAGCGACGGCCGCAGCTTCGATATTTCACTCCATCGCTACAA<br>ATCCACCACCATCATCGAATTCGAGCCCTCGGGCAGCGACGCGCAGCCGCTCGGCACGGC<br>GCGCAAGATGGTGGACCGCATCCGCGAAGCCGACAGCGTCGAAAGCCTGATCTCCAGAAC<br>CACACGCCTGGTAAAGGCGACGCTGGGTTACGACCGGGTGATGATCTACCGCTTCCAGGA<br>AGACGGCGCCGGCAAGGTCGTGTCGGAGGCCAAGCAGCCCGAGCTGGAGAGCTTTCTCGG<br>GCAATATTTCCCCGCCAGCGATATTCCACAGCAGGCTCGCGCACTTTATCTCAAGAACAC<br>CTTGCGCATCATCTCCGATGCCAGCGGCACCCGCATTCCGGTCCTGCCAGCCGTCGATGT<br>CTCCGGTGAGCCGCTCGATCTCTCTTACGCGCATTTGCGCAGCGTCTCGCCCATCCATTG<br>CGAATATCTCCGCAATATGGGTGTTGCCGCCTCCATGTCGATTTCTGTGATTGTCGATGG<br>CGCCCTGTGGGGCCTGATCGCCTGCCACCATTATTCGCCGCGTGTGCTGAGCATGCCAGT<br>TCGGATCGCTGCGGAAATGTTCGGGGAATTTTTCTCCATGCATCTGCAGGTTCTGAAGCA<br>GAAGCGCCGCCTCGACACCATCAACCACGCCCATGCGGCGCTCGACCGGTTTCTGCGGCT<br>GGCCGCCCATCACGCCAACATTGAAGAACTGCTGGTCGACAGTTTTCAGGATTTTGCCGA<br>TCTGATGCCCTGCGACGGTGTCGGGCTGTGGGTCGGCAACAACTGGCACGGCCATGGCGC<br>AACGCCACCGCATGATGCCATTCCAAGGCTGGCGCGTTTCGTCGCTTCGGCTTCGGAAGG<br>CAGGGTCTGGGCAACACACGCCCTGTCGCAGGCCATACCGGAAGCGGAAATATACGCCGG<br>CACCGCGGCCGGAATGCTTGCTATCCCGCTTTCGCAGGTCAAGAGCGACTATCTCCTGTT<br>TTTCCGCAAGGAGATCGTGCAGAACCTGAACTGGGCCGGCAATCCGGAAAAATCCTATGA<br>AACCGGCCCGATGGGCGACCGCCTCACGCCGCGTAAAAGCTTCGCAATCTGGAAAGAGAC<br>CGTTCGCCTGCAGGCACAGCCCTGGTCGGAAGCCGACCGGGAAATCGCCGAAGCCGCGAG<br>AATCGCGCTTGTCGAAGTGGCGTTCCACCACAGCGAGCATCACCATCACCATCACTAAga<br>tccggctgctaacaaagcccgaaaggaagctgagttggctgctgccaccgctgagcaata<br>aCTAGCATAACCCCTTGGGGCCTCTAAACGGGTCTTGAGGGGTTTTTTGctgaaaggagg

**Agp2-PAiRFP2**	cgaaatTAATACGACTCACTATAggGGAATTGTGAGCGGATAACAATTCCcctctagaaa<br>taattttgtttaactttaagAAGGAGatatacatATGGCATCCACGGACTATCATGTCGA<br>CCTGACGAATTGCGACCGCGAACCCATTCATATTCCAGGTTATATTCAACCGCATGGCTG<br>CCTCATCGCCTGCGATAACGCCATGCGTATGGTGCTGCGCCATTCCGAAAACTGCGGCGA<br>ACTGCTGGGTCTCGAGGGCGATCTCAACGGCCGCACCGCCGAAGATGTGCTCGGCAAACG<br>TCTCGTTCATGATCTCCGCAACGCGCTCACCGTTACCGGTAAAACCACGCGTCCCGCCAT<br>GCTGCCGGCAATGGAAACGAGCGACGGCCGCAGCTTCGATATTAGCCTCCATCGCTACAA<br>ATCCACCACCATCATCGAATTCGAGCCCAGCGACAGCGACACGCAGCCGCTCGGCACGGC<br>GCGCAAGATGGTGGACCGCATCCGCGAAGCCGACAGCGTTGAAAGCCTGATCTCCCGTAC<br>CACGCGCCTGGTAAAGGCGACGCTGGGTTACGACCGTGTGCTGATCTACCGCTTCGAAGA<br>AGACGGTGCCGGCAAGGTTGTGAGCGAGGCCAAGCAGCCCGAGCTGGAGAGCTTTCTCGG<br>GCAATATTTCCCCGCCAGCGATATTCCACAGCAGGCTCGCGCACTTTATCTCAAGAACAC<br>CTTGCGCATCATCTCCGATGCCAGCGGCACCCCGATTCCGGTGCTGCCAGCCGTTGATGT<br>TTCCGGTGAGCCGCTCGATCTCTCTTACGCGCATTTGCGCAACTTCAGCCCCATCCATTG<br>CGAATATCTCCGCAATATGGGTGTTGCCGCCTCCATGAGCATTTCTGTGATTGTTGATGA<br>CGCCCTGTGGGGCCTGATCGTTTGCCACCATTGTAGCCCGCGTGTGCTGAGCATGCCAGT<br>TCGTATCGCTGCGGCGATGTTCGGGGAATTTTTCTCCATGTTCCTGCAGGTTCTGAAACA<br>GAAGCGCCGCCTCGACACCATCAACCACGCCCATGCGGCGCTCGACCGTTTTCTGCGCCT<br>GGCCGCCCATCGTGCCAACCTCGAAGAACTGCTGGTTGACAGCTTTCAAGATTTTGCCCG<br>CCTGATCCCCTGCGACGGTGTGGGGCTGTGGGTTGGCAATAACTGGCACGGCCATGGCGC<br>AACGCCACCGCATGATGCCATTCCACGTCTGGCACGTTTCGTTGCTAGCGTTAGCGAAGG<br>CCGTGTTTGGGCAACGCACGCCCTGAGCCAGGCCATCCCGGAAGCGGAAATCTACGCCGA<br>CACCGCTGCCGGTATGCTTGCTATCCCGATTAGCCAGGTTAAGAGCGACTATCTCCTGTT<br>TTTCCGCAAAGAGATCGTGCAGAACCTGAACTGGGCCGGCAATCCGGAAAAATCCTATGA<br>AACCGGCCCGATGGGCGACCGCCTCACGCCGCGTAAAAGCTTCGCAATCTGGAAAGAGTC<br>CGTTCGCCTGCAGGCACAGCCCTGGAGCGAAGCCGACCGCGAAATCGCCGAAACCGCGCG<br>CATCGCGCTTGTTGGTGTGGCGTTCCACCACAGCGAGTTGATGGCAGGTCTCGAGTACAA<br>GCATCACCATCACCATCACTAAgatccggctgctaacaaagcccgaaaggaagctgagtt<br>ggctgctgccaccgctgagcaataaCTAGCATAACCCCTTGGGGCCTCTAAACGGGTCTT<br>GAGGGGTTTTTTGctgaaaggaggaactat

Colour Code: T7-promotor, lac operator, ribosom binding site, coding sequence, T7-terminator

Supplementary References

1. Adams PD*, et al.* PHENIX: a comprehensive Python-based system for macromolecular structure solution. *Acta Crystallographica Section D* **66**, 213-221 (2010).
2. Praznikar J, Afonine PV, Guncar G, Adams PD, Turk D. Averaged kick maps: less noise, more signal...and probably less bias. *Acta Crystallographica Section D* **65**, 921-931 (2009).
3. Liebschner D*, et al.* Polder maps: improving OMIT maps by excluding bulk solvent. *Acta Crystallographica Section D* **73**, 148-157 (2017).
4. Brünger AT, Rice LM. Crystallographic Refinement by Simulated Annealing: Methods and Applications. In: *Methods in Enzymology* (ed^(eds). Academic Press (1997).
5. McDonald IK, Thornton JM. Satisfying Hydrogen Bonding Potential in Proteins. *J Mol Biol* **238**, 777-793 (1994).
6. Laskowski RA, Swindells MB. LigPlot+: multiple ligand-protein interaction diagrams for drug discovery. *J Chem Inf Model* **51**, 2778-2786 (2011).
7. Piatkevich KD, Subach FV, Verkhusha VV. Far-red light photoactivatable near-infrared fluorescent proteins engineered from a bacterial phytochrome. *Nat Commun* **4**, 2153 (2013).
8. Lamparter T, Michael N, Mittmann F, Esteban B. Phytochrome from Agrobacterium tumefaciens has unusual spectral properties and reveals an N-terminal chromophore attachment site. *Proc Natl Acad Sci U S A* **99**, 11628-11633 (2002).
9. Krieger A, Molina I, Oberpichler I, Michael N, Lamparter T. Spectral properties of phytochrome Agp2 from Agrobacterium tumefaciens are specifically modified by a compound of the cell extract. *Journal of Photochemistry and Photobiology B: Biology* **93**, 16-22 (2008).
10. Eilfeld P, and Rüdiger W. Absorption spectra of phytochrome intermediates. *Zeitschrift für Naturforschung C*, **40** (1-2), 109-114. (1985).
11. Mueller-Dieckmann C*, et al.* The status of the macromolecular crystallography beamlines at the European Synchrotron Radiation Facility. *The European Physical Journal Plus* **130**, (2015).
12. Nurizzo D*, et al.* The ID23-1 structural biology beamline at the ESRF. *Journal of Synchrotron Radiation* **13**, 227-238 (2006).
13. Mueller U*, et al.* The macromolecular crystallography beamlines at BESSY II of the Helmholtz-Zentrum Berlin: Current status and perspectives. *The European Physical Journal Plus* **130**, 141 (2015).
14. Velazquez Escobar F*, et al.* A protonation-coupled feedback mechanism controls the signalling process in bathy phytochromes. *Nat Chem* **7**, 423-430 (2015).
15. Buhrke D, Kuhlmann U, Michael N, Hildebrandt P. The photoconversions of phytochromes include an unproductive shunt reaction pathway. *Chem. Phys. Chem.* **19**, 566-570 (2018).
16. Yang X, Ren Z, Kuk J, Moffat K. Temperature-scan cryocrystallography reveals reaction intermediates in bacteriophytochrome. *Nature* **479**, 428-432 (2011).
17. Bellini D, Papiz MZ. Structure of a bacteriophytochrome and light-stimulated protomer swapping with a gene repressor. *Structure* **20**, 1436-1446 (2012).
18. Otero LH*, et al.* Structure of the Full-Length Bacteriophytochrome from the Plant Pathogen Xanthomonas campestris Provides Clues to its Long-Range Signaling Mechanism. *Journal of Molecular Biology* **428**, 3702-3720 (2016).
19. Nagano S*,* Scheerer P, *et al.* The Crystal Structures of the N-terminal Photosensory Core Module of Agrobacterium Phytochrome Agp1 as Parallel and Anti-parallel Dimers. *J Biol Chem* **291**, 20674-20691 (2016).
20. Gourinchas G, Etzl S, Göbl C, Vide U, Madl T, Winkler A. Long-range allosteric signaling in red light–regulated diguanylyl cyclases. *Science Advances* **3**, e1602498 (2017).

21. Burgie ES, Wang T, Bussell AN, Walker JM, Li H, Vierstra RD. Crystallographic and electron microscopic analyses of a bacterial phytochrome reveal local and global rearrangements during photoconversion. *Journal of Biological Chemistry* **289**, 24573-24587 (2014).
22. Woitowich NC, *et al.*, Structural basis for light control of cell development revealed by crystal structures of a myxobacterial phytochrome. *IUCrJ.* **5** (Pt 5), 619-634 (2018).
23. Edgar RC. MUSCLE: multiple sequence alignment with high accuracy and high throughput. *Nucleic Acids Res* **32**, 1792-1797 (2004).
24. Pettersen EF*, et al.* UCSF Chimera—A visualization system for exploratory research and analysis. *Journal of Computational Chemistry* **25**, 1605-1612 (2004).
